# Current-induced dynamics in carbon atomic contacts

**DOI:** 10.3762/bjnano.2.90

**Published:** 2011-12-16

**Authors:** Jing-Tao Lü, Tue Gunst, Per Hedegård, Mads Brandbyge

**Affiliations:** 1 DTU-Nanotech, Dept. of Micro- and Nanotechnology, Technical University of Denmark (DTU), Ørsteds Plads, Bldg. 345E, DK-2800 Lyngby, Denmark; 2 Niels Bohr Institute, Nano-Science Center, University of Copenhagen, Denmark

**Keywords:** carbon-nanoelectronics, current-induced forces, molecular contacts, nanoscale Joule heating, semiclassical Langevin equation

## Abstract

**Background:** The effect of electric current on the motion of atoms still poses many questions, and several mechanisms are at play. Recently there has been focus on the importance of the current-induced nonconservative forces (NC) and Berry-phase derived forces (BP) with respect to the stability of molecular-scale contacts. Systems based on molecules bridging electrically gated graphene electrodes may offer an interesting test-bed for these effects.

**Results:** We employ a semi-classical Langevin approach in combination with DFT calculations to study the current-induced vibrational dynamics of an atomic carbon chain connecting electrically gated graphene electrodes. This illustrates how the device stability can be predicted solely from the modes obtained from the Langevin equation, including the current-induced forces. We point out that the gate offers control of the current, independent of the bias voltage, which can be used to explore current-induced vibrational instabilities due the NC/BP forces. Furthermore, using tight-binding and the Brenner potential we illustrate how Langevin-type molecular-dynamics calculations including the Joule heating effect for the carbon-chain systems can be performed. Molecular dynamics including current-induced forces enables an energy redistribution mechanism among the modes, mediated by anharmonic interactions, which is found to be vital in the description of the electrical heating.

**Conclusion:** We have developed a semiclassical Langevin equation approach that can be used to explore current-induced dynamics and instabilities. We find instabilities at experimentally relevant bias and gate voltages for the carbon-chain system.

## Introduction

The effects of electric current on the motion of atoms have become particular important in the on-going quest for molecular-scale electronics [1–4]. Atomic motion due to electric current is behind the long-term breakdown of interconnects leading to failure in integrated circuits. This effect is of even greater importance for systems where the bottle-neck for the current flow is a few chemical bonds. The inelastic scattering of electrons by atomic vibrations leads to the well-known effect of Joule heating, which can have an impact on the electrical behavior and stability. However, recently it was pointed out [5–8] that other current-induced forces can play a role. For instance, in the case of molecular contacts with conductance on the order of *G*_0_ = 2*e*^2^/*h* = 1/12.9 kΩ (*e* being the electron charge and *h* Planck’s constant), and under “high” bias voltage (~1 V), the current-induced forces that do not conserve the energy of the atomic motion may lead to run-away behavior. However, experiments in this regime are very challenging. For example, for the typical experiments involving molecular-scale contacts between bulk electrodes it is not possible to image the atomic structure while the contacts are in place and current is flowing. Furthermore, it is far from being trivial to add additional gate potentials in order to modify the electronic structure and gain independent control of the bias voltage and current [3,9].

On the theoretical side, it is desirable to develop computer simulation techniques, such as molecular dynamics (MD), preferably without adjustable parameters, to study in detail the complex current-driven atomic processes. To this end, we recently developed an approach based on the semiclassical Langevin equation, which may form the basis of MD. In this description the nonequilibrium electronic environment is described as an effective “bath” influencing the atomic dynamics. In particular, we identified the forces acting on the atoms due to the electric current. These include “extra” fluctuating forces yielding the Joule heating, a nonconservative “electron-wind” force (denoted NC), recently discussed by Todorov and co-workers [5], and a Lorentz-like force originating from the quantum-mechanical “Berry phase” of the electronic subsystem [6] (denoted BP). The purpose of this article is two-fold. We will illustrate this semiclassical Langevin approach and show how the current-induced effects could be investigated in molecular contacts connecting gated graphene or nanotube electrodes.

Graphene is now being explored very extensively due to its outstanding electrical and thermal transport properties [10–12]. Besides being highly important in their own right, carbon nanotube- or graphene-based nanostructures may offer an interesting test bed for studies of current-induced effects at the atomic scale. For such systems, experiments with atomic resolution, employing for instance state-of-the-art electron microscopes, can be performed in the presence of current, allowing the dynamics to be followed down to single adatoms [13]. Electric current has been used to induce changes in graphene-edges, which were monitored while a current was simultaneously passed through the structure [14]. This was explained as carbon edge-dimers desorbing due to Joule-heating [15]. Taking this a step further, one can imagine that nanostructured nanotubes or graphene can be used as an electrode interface to molecular devices based on organic chemistry [16]. Especially promising aspects include the inherent 2-D geometry of graphene, which enables both straightforward electrical gating, and atomic-scale imaging in the presence of current. There have been a number of microscopy studies of single-atom carbon chains bridging graphene [13,17] or nanotubes [18]. On the theoretical side, various aspects of these systems have been studied, such as the formation of chains [19–20], their stability [21], and electron-transport properties [22–24]. Here we explore the current-induced forces and nanoscale Joule heating of the carbon chain system between electrically gated graphene electrodes.

The paper is organized as follows. After a brief outline of the semiclassical Langevin method, we will use it to study the dynamics of the carbon chain as a function of bias and gate voltages. We point out that the gate, which offers independent control of bias voltage and current in the system, can be used to explore current-induced vibrational instabilities in the current-carrying chain. Finally, we illustrate how the Langevin molecular dynamics can be performed for a carbon-chain system with the Joule heating effect included, by using tight-binding and the Brenner potential.

## Results and Discussion

### Semiclassical Langevin dynamics

We outline the Langevin approach here. For a classical oscillator system (mass-scaled coordinate *x*) in a general nonlinear force-field, *F*, coupled linearly to a *bath* of harmonic oscillators, it is possible to eliminate the bath variables and describe the system using the generalized Langevin equation, [25–27],

[1]
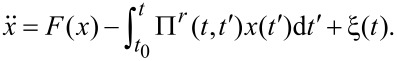


Here the bath influences the motion through two distinct force contributions, (i) a retarded time-kernel, Π*^r^*, describing the back-action at time *t* after propagation in the bath due to the motion of *x* at an earlier time, and (ii) a force ξ of statistical nature originating from the thermal fluctuations of the bath. In the case of thermodynamic equilibrium, ξ is characterized by a temperature and is related to Π*^r^* by the fluctuation-dissipation theorem. Note that in general *x*, *F*, and ξ are vectors and Π*^r^* is a matrix. This method was used by Wang and co-workers [28–29] to describe thermal transport in the quantum limit, with phonons in the two connecting reservoirs with different temperature acting as baths and with their quantum fluctuations included in ξ. This reproduced the Landauer result of thermal transport in the harmonic case [28].

It is possible to reach a semiclassical Langevin equation description of the motion of the ions coupled to the electron gas if we assume a linear coupling to the electronic environment: Either in the displacement from an equilibrium or in the velocity (adiabatic expansion) of the ions. This Langevin/Brownian motion approach to atomic scattering at metal surfaces has a rather long history in the case of metal electrons in thermal equilibrium [30–31].

We have extended this to describe the dynamics of the ions in a nanoconductor between metal electrodes in the nonequilibrium case, where an electric current is present [6,32]. In order to sketch the derivation, we consider a displacement-dependent tight-binding model with electron states in the scattering region of interest *k,l*, and with *H_el_* being the static electronic Hamiltonian (scattering region and its coupling to the left and right electrodes [33]),

[2]
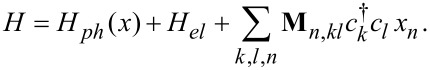


Here *x* is a column vector comprising the mass-normalized displacement operators for each degree of freedom, e.g., 
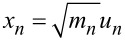
, *u_n_* and *m_n_* being the displacement operator and mass, and *H_ph_* = 1/2
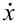
^T^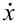 + 1/2*x*^T^*Kx* corresponds to a set of harmonic oscillators that couple with the electrons, *K* being the dynamical matrix. We construct a localized basis-set describing the electrons in the scattering region, where 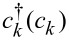 is the electron creation (annihilation) operator at site *k* in this region [34]. Here we only consider the coupling to the electron bath, but the linear coupling to an external phonon bath can be taken into account along the same lines and adds a contribution to Π*^r^*. The derivation and result for a linearly coupled harmonic phonon bath is similar, and was given in [28]. Alternatively, the dynamics of some external phonons, not coupling to the electrons directly, may be treated explicitly in actual MD calculations, as we illustrate below (regions *DL, DR* in Figure 6a). The electron–phonon coupling corresponds to matrix elements of the force operator **M***_n,kl_* = 
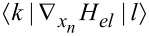
. We assumed that **M** is small by keeping only the term that is linear in *x*.

We may obtain an equation of motion for *x* using Heisenberg’s equation of motion, 
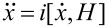
, based on atomic units (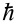 = 1) and implicit mode index (*n*),

[3]
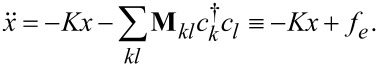


The term 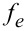 describes the “forces” due to the interaction with the electron gas. Importantly, these forces are random in nature [35]. We can calculate the mean value of 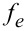 by averaging it over the nonequilibrium electronic state,

[4]
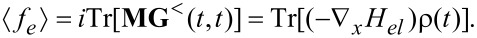


Here we introduce the electrical lesser-Green’s function, 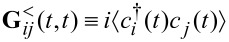, which is equivalent to the density matrix, ρ (multiplied by −*i*), and depends on *x*(*t*), since the electrons are coupled to *x* in the Hamiltonian. This is similar to the expression for the average force in Ehrenfest dynamics [5].

We can evaluate this perturbatively by using the unperturbed-electron lesser Green’s function, 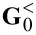, corresponding to the case of steady-state electron transport without electron–phonon interaction [33],

[5]
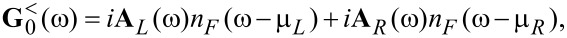


where **A***_L_*_/_*_R_* are the density of state matrices for electronic states originating in the left/right electrodes, each with chemical potential μ*_L_*_/_*_R_* [33], which differ for finite bias voltage, *V*, as μ*_L_* − μ*_R_* = *eV*, and *n_F_*(ω) = 1/(*e*^ω/^*^k^_B_^T^* + 1) is the Fermi-Dirac distribution function. We thus treat the nonequilibrium electron system as a reservoir unperturbed by the phonons. Using the nonequilibrium Greens function (NEGF) technique [36], we may write the 2nd lowest orders in **M** of 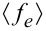 as,

[6]
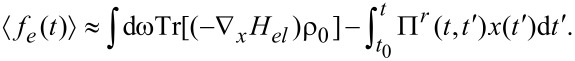


The first term yields a constant force due to the change in electron bonding with bias and a “direct force” due to interaction of charges with the field [37]. Here ρ_0_ = ρ_eq_ + δρ is the nonequilibrium electron-density matrix *without* electron–phonon interaction. We split it into an equilibrium contribution ρ_eq_ and a nonequilibrium correction δρ. In linear response, we obtain a term 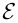 · *x* from the field in *H_el_*, 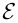 being the external field, which yields a “direct” force involving the equilibrium ρ_eq_. We also obtain a term involving *H_el_*(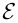 = 0), together with the change in density to first order in the field 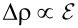, in the first term of Equation 6, resulting from the change of density in the chemical bonds due to the current [38–39].

The second contribution is the retarded back-action of the electron gas due to the motion and is equivalent to the retarded phonon self-energy. In the steady state, Π*^r^* only depends on the time difference, and it is convenient to work in the frequency (energy) domain. This can be expressed by using the coupling-weighted electron–hole-pair density of states, Λ^αβ^, inside or between electrodes α,β 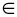 *L,R*,

[7]
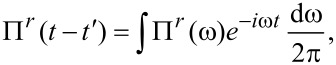


[8]
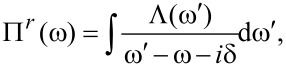


where Λ can be expressed in terms of the electrode DOS,

[9]
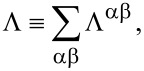


[10]
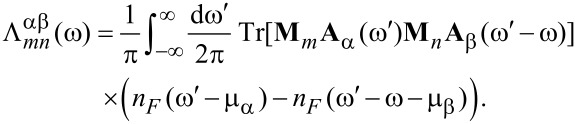


We have included a factor of 2 from the spin degeneracy and have explicitly included the mode index, *m,n* on the coupling matrices, **M**, and on Λ in Equation 10.

The forces described by 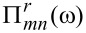 in Equation 6 contain a number of interesting current-induced effects. It is instructive to split the kernel into parts,

[11]
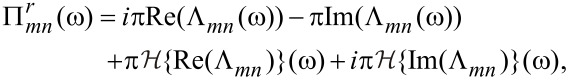


where


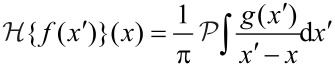


is the Hilbert transform. The Λ matrix has the following symmetry properties when exchanging modes(*n* ↔ *m*) and electrodes(α ↔ β),

[12]
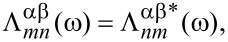


and

[13]
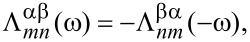


which are helpful when examining the terms in Equation 11, which are summarized in the following:

- **Friction** – The first term in Equation 11 is imaginary and symmetric in mode index *m,n*. It describes the friction force due to the generation of electron–hole pairs in the electronic environment by the ionic motion. This process exists even in equilibrium [31]. For slowly varying **A***_L_*_/_*_R_* with energy as compared to the vibrational energies (wide-band limit) we obtain the simple time-local electron friction force, 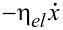, with

[14]
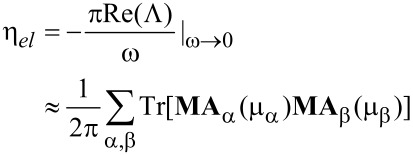

- **NC (wind) force** – The second term in Equation 11 is real and antisymmetric, which means that the general curl of this force is not zero. It describes the NC force, discussed very recently by Dundas and co-workers [5]. This force is finite, even in the limit of zero frequency, where the friction and Joule heating effect is not important anymore.
- **Renormalization** – The third term is real and symmetric and can be interpreted as a renormalization of the dynamical matrix. It contains an equilibrium part and a nonequilibrium correction. The equilibrium part is already included in the dynamical matrix when we calculate it within the Born–Oppenheimer approximation. The nonequilibrium part gives a bias-induced modification of the harmonic potential.
- **BP force** – Finally, the last term is imaginary, antisymmetric, and proportional to ω for small frequencies. It can be identified as the “Berry phase” (BP) force in [6]. Since the direction of this force is always normal to the velocity in the abstract phase space, it does no work, resembling a Lorentz force with effective magnetic field

[15]
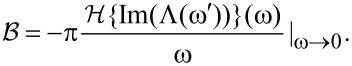

- **Random forces** – The randomness of the force 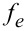 is characterized by its correlation function in the frequency domain, which can again be calculated with NEGF. However, we note that since 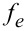 is a quantum operator, 
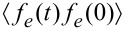
 does not result in a real number. Instead we use the symmetrized and real 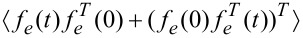. This expression equals the semiclassical result obtained from the path-integral derivation of the Langevin equation [6,35] and reads, in Fourier space,

[16]
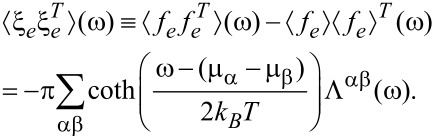

- This spectral power density can be used to generate an instance of the Gaussian random noise as a function of time that is needed in MD simulations. Most importantly this random force contains not only the thermal excitations but also the excess excitations leading to Joule heating [32], through the dependence of the chemical potentials μ*_L_* − μ*_R_* = *eV*. Thus with this formalism it is possible to disentangle the various contributions to the forces, being either deterministic or random in nature.

### Current-induced vibrational instability

**Figure 1:**
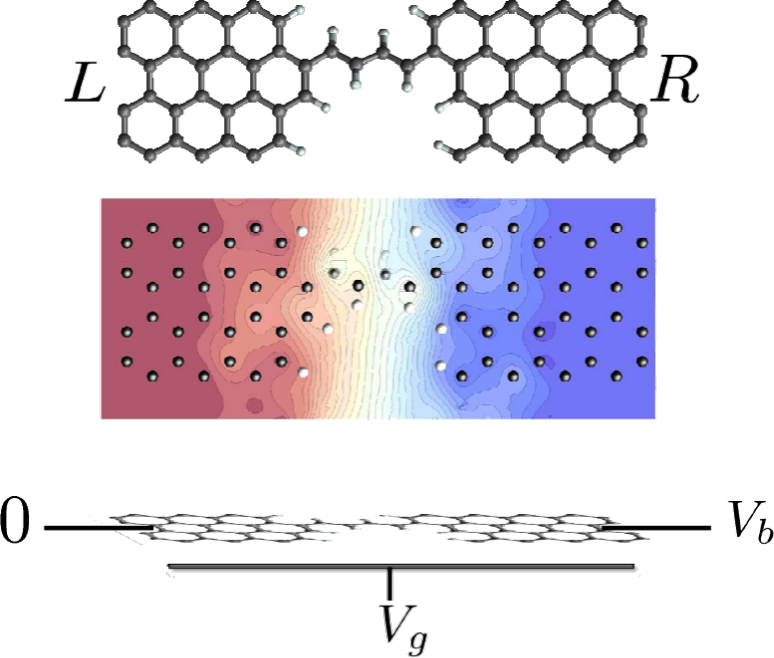
The system considered in the present study is a four-atom carbon chain bridging two graphene electrodes. The dangling bonds are passivated by hydrogen atoms. In addition to the bias applied between the left (*L*) and right (*R*) electrodes (*V_b_*), a gate potential (*V_g_*) can also be applied perpendicular to the graphene surface. The center panel shows the calculated contour plot of the electrostatic-potential drop across the junction at *V_g_* = 0 V, and *V_b_* = 1 V. The equal drop at the left and right electrodes reflects the electron–hole symmetry for *V_g_* = 0 V [40].

**Figure 2:**
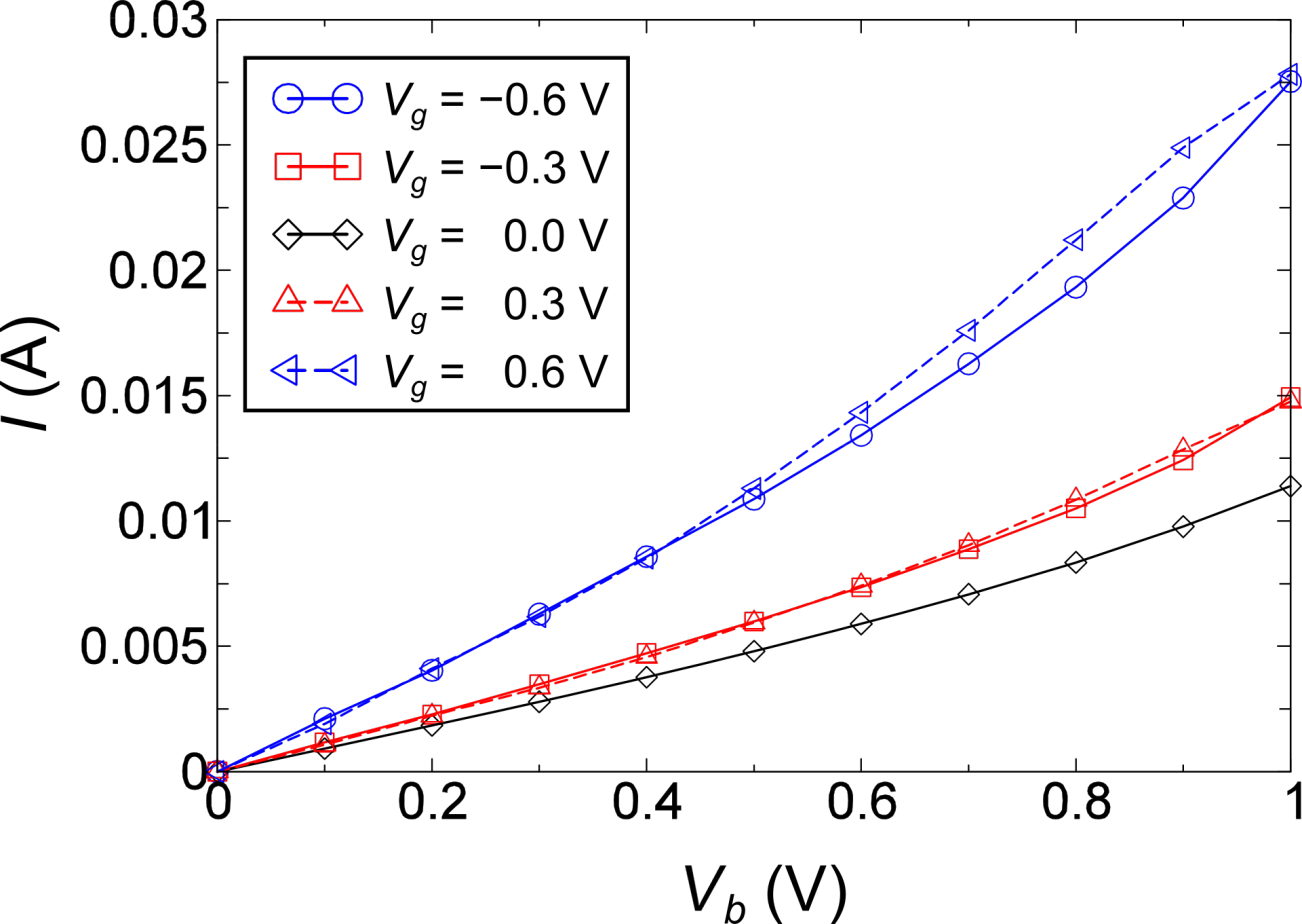
Current–Voltage (*I*−*V_b_*) curves at different *V_g_*.

We now turn to illustrations of the use of the semiclassical Langevin equation to describe current-induced effects. In this section we employ it to study the effect of the current-induced forces and Joule heating on the stability of the system, within the harmonic approximation. We will here ignore the coupling to electrode phonons. This makes an eigen-mode analysis possible, which eases the interpretation of the results. The model system we use is shown in Figure 1, in which a four-atom carbon chain is bridged between two graphene electrodes (*L* and *R*). We assume a field effect transistor setup, in which a gate potential, *V_g_*, is applied to the system in addition to the bias applied between the two electrodes, *V_b_*. We will show that this offers a convenient way to explore current-induced vibrational instabilities. We can already see the effect of the gate potential in the current–voltage (*I* − *V_b_*) characteristics shown in Figure 2.

**Figure 3:**
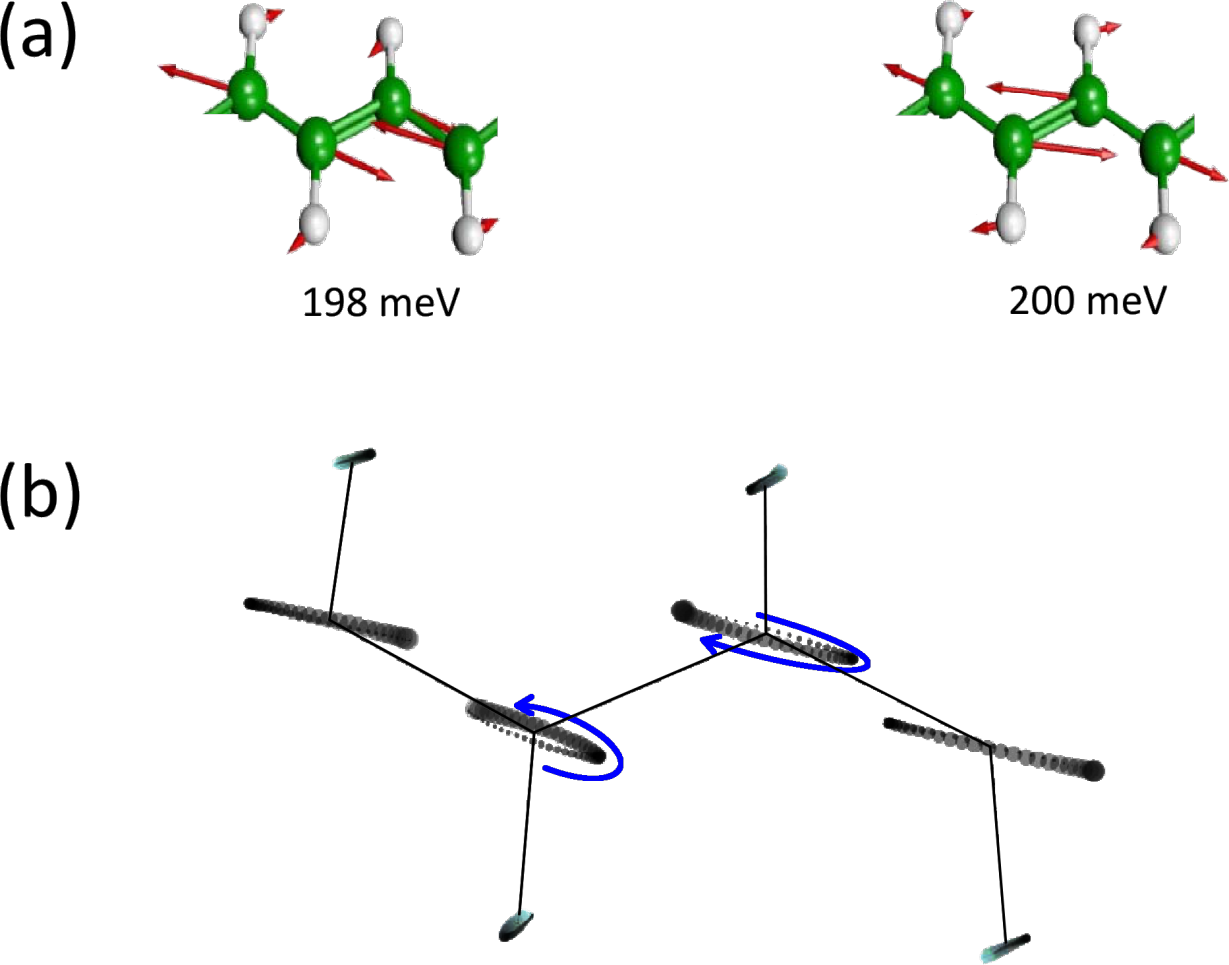
(a) Motion of the two phonon modes around 200 meV. (b) Motion of the runaway mode at *V_g_* = 0.6 V, and *V_b_* = 1 V. We depict the motion using a number of discrete time steps roughly corresponding to a full period. The position of each atom is depicted as a circle for a sequence of time steps indicated by an increasing radius with time. The motion is a phase-shifted linear combination of the two modes in (a). We can see the elliptical motion of the carbon atoms from the plot. The enclosed area indicates that work can be done by the current-induced NC force.

The effect of the NC and BP forces is to couple different phonon modes with nearly similar frequencies. From now on, we will focus on the two phonon modes around 200 meV, shown in Figure 3, since the alternating-bond-length-type modes (200 meV) couple most strongly with the electric current. This type of mode also gives rise to the most intensive Raman signals in unpassivated chains between graphene-like pieces [41].

The calculation was performed by using the SIESTA density-functional theory (DFT) method [42], which has been extended to study elastic [33] and inelastic [34] transport in molecular conductors. We used similar parameters as detailed in [34], and in order to keep the calculation simple and tractable, we modeled the electrodes by simply employing the Γ k-point in the transverse electrode direction. The electron–phonon coupling matrix (**M**) was calculated at zero bias, whereas we calculated the electronic structure at finite bias. We note that the voltage dependence of the coupling matrix could play a role, but this is beyond the scope of the present more illustrative purpose [43]. Based on these approximations, we can calculate the full ω-dependent Λ function, and the self-energies, Π*^r^*. To perform the eigen-mode analysis, we further assumed linear ω-dependent friction, Berry force (BP), constant nonconservative force (NC), and ignore the renormalization of the dynamical matrix.

We model the effect of *V_b_* as a shift of the equilibrium chemical potential, *E*_F_. In this way we can tune the electronic structure within the bias window by changing the gate potential. In the following, we look at the bias and gate dependence of the inverse Q-factor (1/*Q*) and effective phonon number *N*. The inverse Q-factor for mode *i* (note we use index *i* for full modes including the current-induced forces) is defined as

[17]
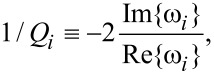


where ω*_i_* are the eigenvalues of the full dynamical matrix, including the current-induced forces. These modes thus consist of linear combinations of the “unperturbed” normal modes of the system, *n,m*, as calculated by using the standard Born–Oppenheimer approximation. The phonon number can be calculated from the displacement correlation function,

[18]
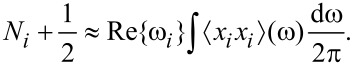


**Figure 4:**
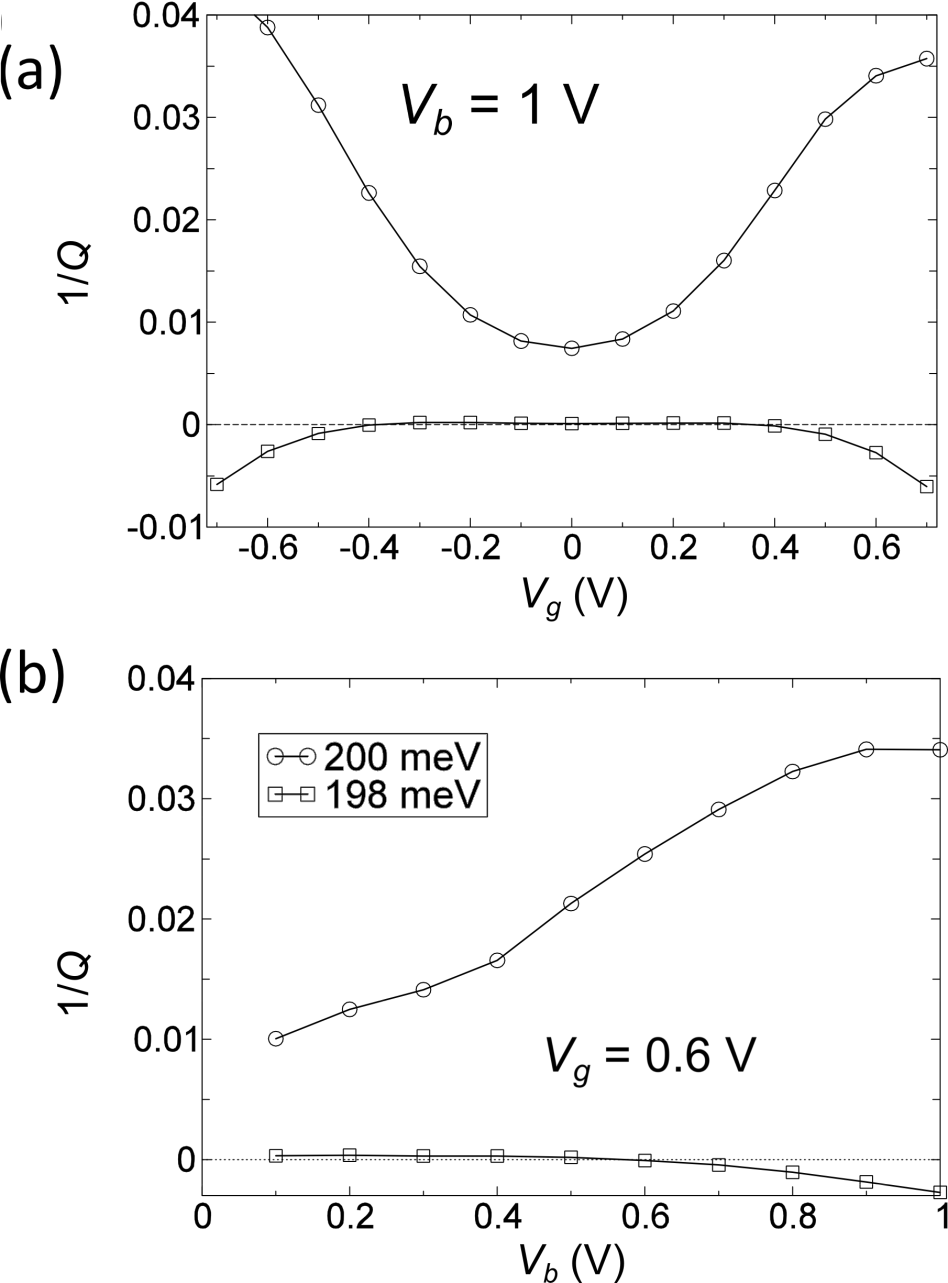
(a) Inverse Q-factor (1/*Q*) as a function of gate voltage, *V_g_*, at *V_b_* = 1 V for the two modes around 200 meV. (b) 1/*Q* as a function of bias voltage, *V_b_*, at fixed gate voltage *V_g_* = 0.6 V, for the same pair of phonon modes.

**Figure 5:**
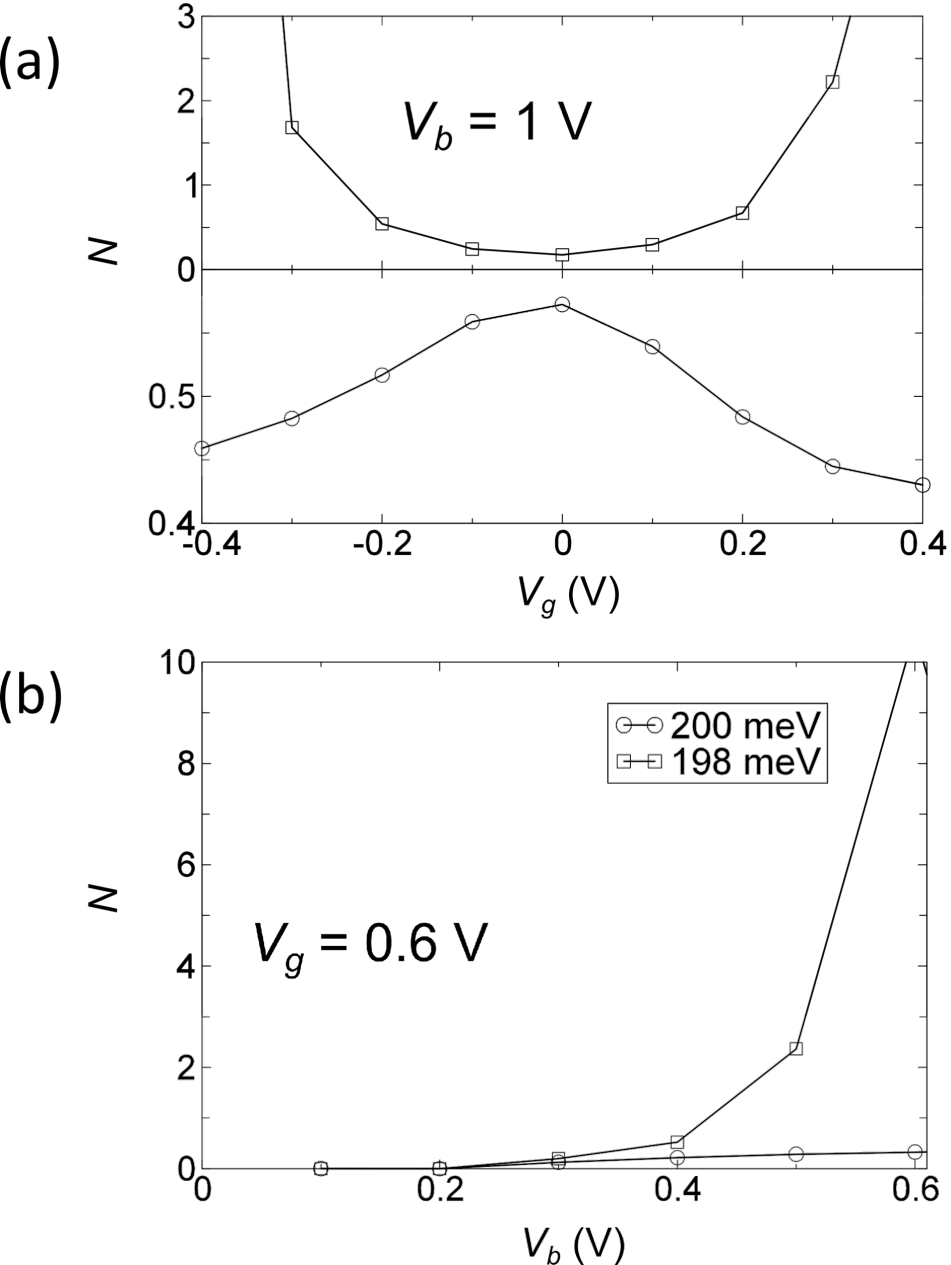
(a) Effective phonon number (*N*) for the two phonon modes around 200 meV as a function of gate voltage, *V_g_*, at fixed bias voltage, *V_b_* = 1 V. (b) *N* as a function of bias voltage, *V_b_*, at fixed gate voltage *V_g_* = 0.6 V. Note that it diverges at the critical point when the damping (1/*Q*) in Figure 4 goes to zero.

We show the bias and gate potential dependence of the inverse Q-factor and phonon number in Figure 4 and Figure 5. The coupling of these two modes due to the bias (gate) dependent NC and BP force changes their lifetime. The two modes always have opposite dependence. The vibrational instability occurs at the critical point where 1/*Q* = 0 around *V_g_* = ±0.4 V. This corresponds to an infinite phonon number in Figure 5, and we therefore call it a “runaway” mode. The motion of this mode at *V_b_* = 1 V, *V_g_* = 0.6 V is plotted in Figure 3b. We can observe the elliptical motion of several atoms in real-space. This is critical because in order for the nonconservative force to do work on the atoms their motion has to enclose a finite area, either in real or in abstract phase space.

Finally, we should mention that when the current hits the instability threshold it will drive the system into a highly anharmonic regime, where the preceding eigenanalysis breaks down. One scenario is that the motion of the system will reach a limit-cycle determined by the detailed anharmonic potential and the interaction with the current [7]. In this regime the details of the damping due to the coupling with phonons in the electrodes could be important, and the electron–phonon coupling could also change from the value given around the harmonic equilibrium position. In order to address this regime we can perform molecular-dynamics simulations, taking into account both the coupling between different modes and their coupling with the electrode phonons, in order to study how the system actually reacts due to the instability.

### Molecular dynamics with Joule heating

**Figure 6:**
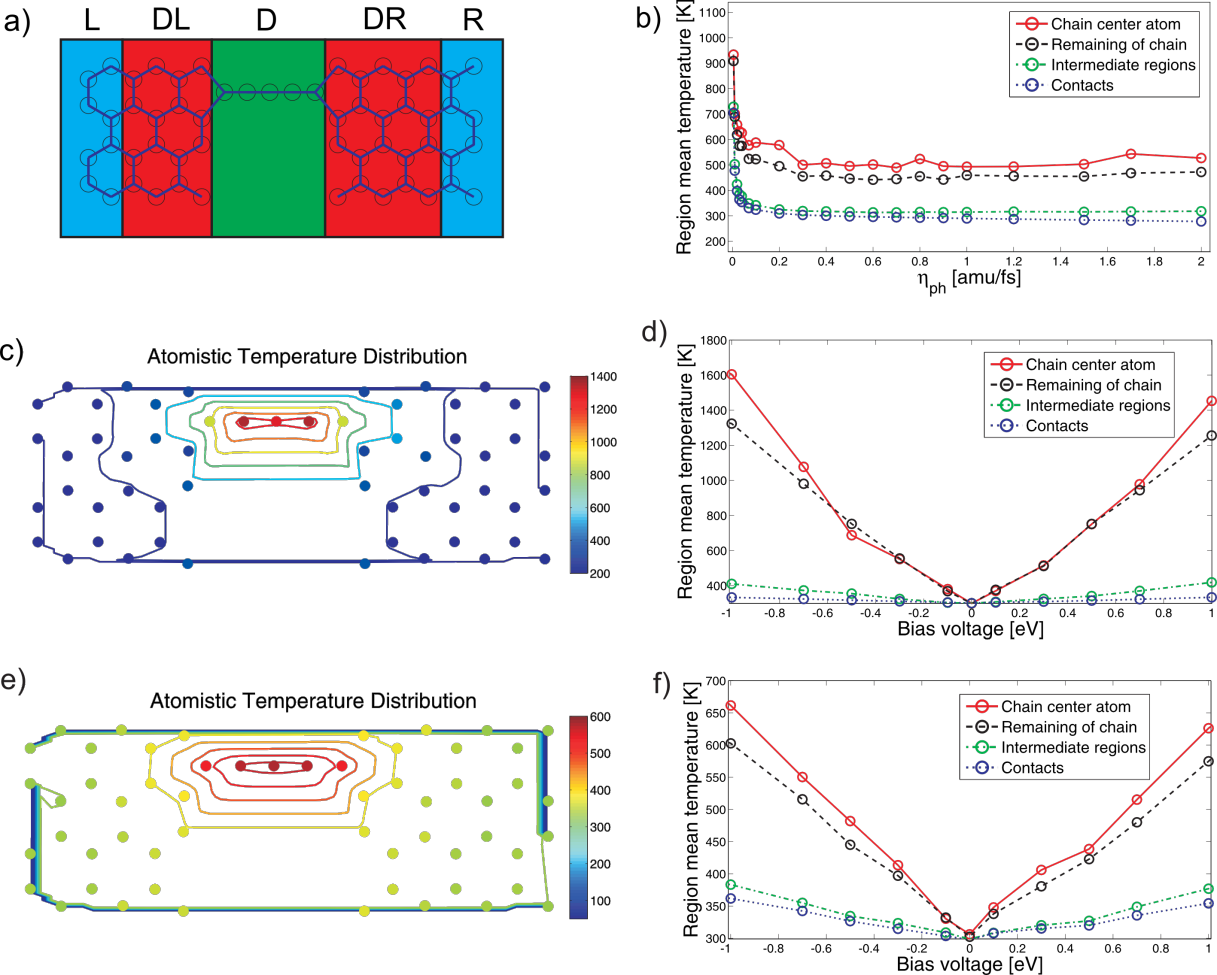
(a) Definition of the system regions with different types of noise contributions. Leads (*L,R*) have a well-defined temperature determined by the phonon noise, the device (*D*) temperature is defined from the electrical heating, and the intermediate regions (*DL,DR*) are free and are heated by propagation noise. In the MD setup no atoms are held fixed, but periodic boundary conditions are applied. The figure describes the setup in which the local temperatures plotted in (c) and (e) should be understood. (b) Temperature of the regions as a function of phonon friction. (c, d) Obtained temperatures at different atoms within the harmonic approximation. (c) The simulations were run at T = 300 K and at *eV_b_* = 1 eV, and (d) varying bias voltages. (e, f) Corresponding atomistic temperature distributions including the anharmonic interactions. The lead temperature can exceed the equilibrium bath temperature due to propagation noise. In particular, the anharmonic interactions redistribute part of the energy from the modes in the chain to the bulk modes in the lead.

Next we illustrate the use of the Langevin equation to perform molecular-dynamics simulations of a carbon-chain system, in the presence of current flow, in the simplest possible setting, but now including coupling to the electrode phonons. Therefore we abandon the DFT approach, and instead employ the widely used π-tight-binding model with hopping parameter β = 2.7 eV, and the Brenner potential for calculations of the interatomic forces [44]. We consider the unpassivated structure in Figure 6. The electron–phonon coupling is modeled by the Harrison scaling law [45], β = 2.7eV (*a*_0_/*d*)^2^, determining how β is modified when the nearest neighbor distance, *d*, is changed from the equilibrium value, *a*_0_ = 1.4 Å. The same model has recently been applied in the study of the effect of strain on the electronic structure of graphene [46]. In the simulation we model the coupling to the electrode phonons by a friction parameter, η*_ph_*, and a corresponding white equilibrium phonon noise 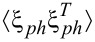 = 2η*_ph_k_B_T* on the *L,R*-electrode regions. This is similar to the stochastic boundary conditions [27] in which *L,R*-atoms act as a boundary. The setup for the MD is shown in (Figure 6a). We include electrode regions that have no interaction with the current (*DL,DR*), and a device region (*D*) where the current density is highest and where the nonconservative forces and Joule heating are included.

Furthermore, instead of using the full nonlocal time-kernel for the electrons in Equation 14, we use the wide-band approximation, and neglect the off-diagonal elements of the electron-noise spectral power density, 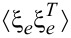
(ω). The diagonal of the electron spectral power can be approximated by white noise in the high-bias and wide-band limits, where variations in the electronic DOS are neglected [47]. The assumption of a white-noise spectrum implies neglect of the equilibrium zero-point motion of the atoms, but most importantly here, it includes the Joule heating effects,

[19]
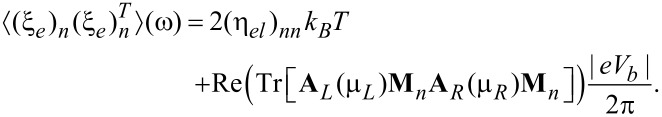


A factor of 2 should be included in the case of spin degeneracy. Based on the velocity Verlet algorithm [48] we carried out MD simulations at a varying bias voltage for zero gate bias (*V_g_* = 0 V), and phonon friction, η*_ph_*. The MD results are summarized in Figure 6b–f. We note that for the present system setup the nonconservative force is found not to play a dominant role compared to the effect of Joule heating. The main insight we gain from the MD example here is that the anharmonic couplings are important and effective in redistributing the energy supplied by the nonequilibrium electrons.

The approximate local phonon friction, η*_ph_*, can in general be expressed from the slope of the corresponding phonon self-energy at zero frequency, as for electrons, see Equation 14. However, here we simply varied its value around this in order to quantify the dependence of the local electrical heating in the device region on this parameter (Figure 6b). The electrical heating of the chain was found not to depend much on the phonon friction when this was chosen to be sufficiently high. This is an appealing result, since it indicates that the electrical heating does not depend critically on the measurement setup, but mainly on the nature of the actual constriction. This seems to be true as long as the heat flow away from the contacts is sufficient to maintain the temperature of the heat baths, and the chain acts as a bottleneck for the heat conduction. However, we note that for heat conduction in the quantum limit it is important to go beyond the white band approximation and include realistic self-energies for the *L,R*-electrode phonons [49]. This will be explored in future work.

Inspired by the equipartition theorem, we define a local temperature variable for the atoms (indexed by *a*) with mass, *m_a_*,

[20]
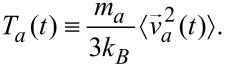


A comparison of the obtained temperature distributions with (Figure 6c, Figure 6d) and without (Figure 6e, Figure 6f) the anharmonic interactions shows that anharmonic couplings between the vibrational modes have a significant influence on the heat-transport properties and local Joule heating of the system. The heating is less localized in the chain due to anharmonicity. This originates from the coupling between different modes and an increased coupling to the surroundings for configurations in which the atoms are displaced from their equilibrium positions. Modes localized in the chain can be heated up to very high temperatures in the harmonic approximation. When anharmonic interactions are included the energy is redistributed and the modes are collectively heated up.

The electron–phonon interaction is typically included through a Taylor expansion of the electronic Hamiltonian around the equilibrium positions (Equation 2). Within the time-local white-noise approximation it is possible to address the effect of changes of electronic Hamiltonian and, especially, electron-phonon coupling on the motion, which was both included in the nonequilibrium force calculations here. This amounts to updating the nonconservative force, friction and noise on the fly along the path. This is possible for the simple parametrization used here. Our preliminary results based on this approximation show that the extra noise contribution from the higher-order couplings may significantly influence the results and increase the electronic heating compared to the static electronic structure approximation. A method which goes beyond white noise and includes the change in electron–phonon coupling when the system is far from the equilibrium positions, e.g., close to bond breaking, remains a challenge for the future.

## Conclusion

We have developed a semiclassical Langevin equation approach, which can be used to explore current-induced atomic dynamics and instabilities in molecular conductors. The Langevin approach can be solved in the harmonic approximation to obtain eigenmodes and their excitation in the presence of current, as well as used for molecular-dynamics simulations based on the full anharmonic potential. Our simple, approximate MD simulation indicates that anharmonic couplings play an important role for the energy redistribution and effective heat dissipation to the electrode reservoirs. However, the MD is computationally very demanding beyond simplified model electronic structures and interatomic potentials, and further developments are necessary. We have used carbon-chain systems both to illustrate the Langevin approach, and in order to highlight how graphene might offer a unique test bed for research into current-induced dynamic effects. Especially, it is straightforward to employ a gate potential to the gate electrode, and thereby obtain independent control of current and bias voltage in the system. Furthermore, atomic-scale resolution can be obtained in electron microscopes in the presence of current, and Raman spectroscopy can give insights into the excitation and effective temperature originating from the electric current [50–52]. Our results for the simplified carbon-chain systems indicate that it may be possible to tune the current-induced instabilities in the atomic dynamics with gate and bias voltages in the experimentally relevant range.
